# Chair side measuring instrument for quantification of the extent of a transverse maxillary occlusal plane cant

**DOI:** 10.1186/s40902-019-0204-6

**Published:** 2019-05-15

**Authors:** Farhad B. Naini, Ashraf Messiha, Daljit S. Gill

**Affiliations:** 1 Kingston and St George’s Hospitals and St George’s Medical School, London, UK; 2 Maxillofacial Unit, St George’s Hospital Medical School, Blackshaw Road, London, SW17 0QT UK; 3 Department of Orthodontics, Great Ormond Street Hospital & Eastman Dental Hospital, London, UK

**Keywords:** Transverse cant, Occlusal plane, Orthognathic surgery, Symmetry

## Abstract

**Background:**

Treatment planning the correction of a transverse maxillary occlusal plane cant often involves a degree of qualitative “eyeballing”, with the attendant possibility of error in the estimated judgement. A simple chair side technique permits quantification of the extent of asymmetry and thereby quantitative measurements for the correction of the occlusal plane cant.

**Methods:**

A measuring instrument may be constructed by soldering the edge of a stainless steel dental ruler at 90° to the flat surface of a similar ruler. With the patient either standing in natural head position, or alternatively seated upright in the dental chair, and a dental photographic retractor in situ, the flat under-surface of the horizontal part of this measuring instrument is placed on a unilateral segment of a bilateral structure, e.g. the higher maxillary canine orthodontic bracket hook. The vertical ruler is held next to the contralateral canine tooth, and the vertical distance measured directly from the canine bracket to the flat under-surface of the horizontal part of the measuring instrument.

**Results:**

This vertical distance quantifies the overall extent of movement required to level the maxillary occlusal plane.

**Conclusions:**

This measuring instrument and simple chair side technique helps to quantify the overall extent of surgical levelling required and may be a useful additional technique in our clinical diagnostic armamentarium.

## Background

Relative unilateral vertical over- or underdevelopment of the maxilla and maxillary dentoalveolus leads to a transverse cant of the maxillary occlusal plane [1]. Correction of such a cant requires a Le Fort I level osteotomy, followed by unilateral bone removal and superior repositioning, contralateral inferior repositioning and bone grafting, or a combination of the two, in order to level the maxillary occlusal plane [2]. The degree of unilateral superior versus contralateral inferior repositioning depends on the aesthetic parameters of maxillary incisor exposure in repose and overall maxillary dentogingival exposure on smiling [1, 2]. Accurate planning to correct such an asymmetry is paramount [3–6]. The following instrument and chair side technique to quantify the extent of asymmetry have not been previously described.

## Methods

**Fig. 1:**
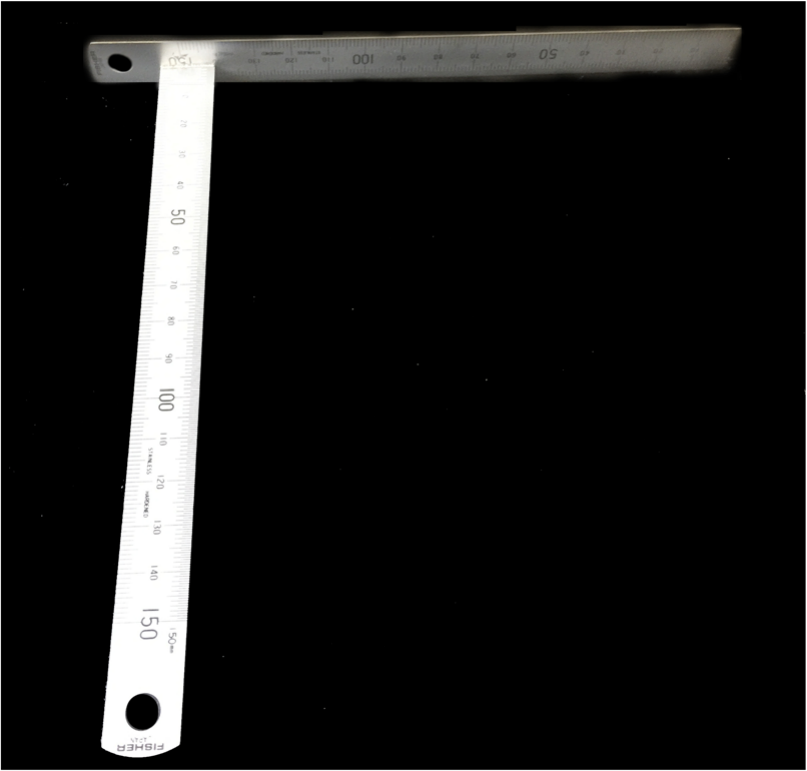
The measuring instrument is constructed by soldering the edge of a double-sided stainless steel dental ruler at 90° to the flat surface of a similar ruler. A double-sided ruler permits its use on the patient’s right and left sides as required. A right-angle gauge may be used to ensure a 90° angle

**Fig. 2:**
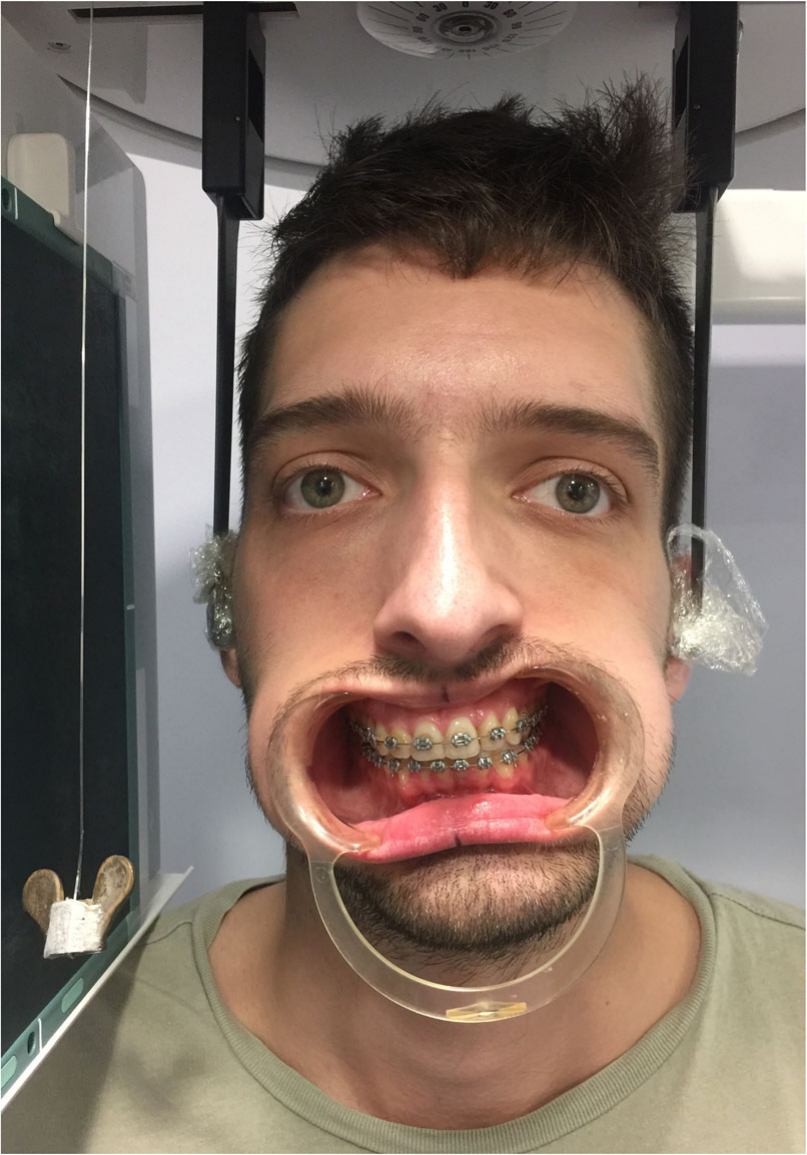
Patient is shown in natural head position. The oral retractors are in situ, and a plumb line is evident hanging to the patient’s right side, which acts as a guide to the true vertical line. A transverse cant of the maxillary occlusal plane, down on the patient’s right side, is evident. The patient may be positioned in a cephalostat as demonstrated here, though this is not mandatory, and for most patients sitting in the dental chair will suffice

**Fig. 3:**
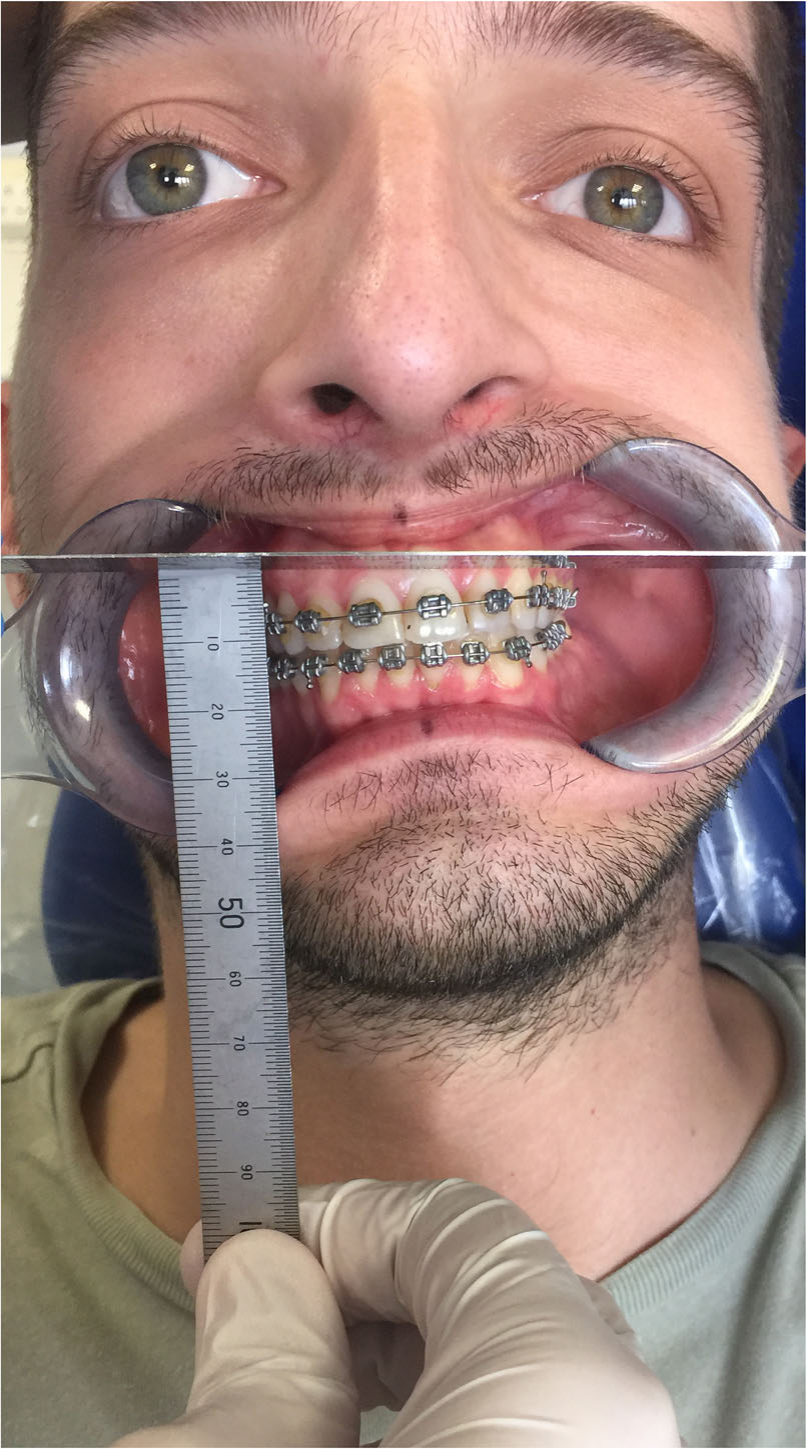
The flat under-surface of the horizontal part of the measuring instrument is placed on the higher maxillary canine orthodontic bracket hook (though it may be placed on a unilateral segment of any bilateral structure), and held perpendicular to the true vertical. For patients with a relatively symmetrical upper face, and no vertical orbital dystopia, the horizontal part of the measuring instrument may be held approximately parallel to the interpupillary plane (a dental mirror handle may be held in line with the interpupillary plane, to aid visualisation at the chair side). The vertical ruler is held next to the contralateral canine tooth, and the vertical distance measured directly from the canine bracket to the flat under-surface of the horizontal part of the measuring instrument. This vertical distance quantifies the overall extent of movement required to level the maxillary occlusal plane

A measuring instrument may be constructed by soldering the edge of a double-sided stainless steel dental ruler at 90° to the flat surface of a similar ruler (Fig. 1). With the patient in natural head position, near a vertical plumb line (forming a true vertical line), and a dental photographic retractor in situ (Fig. 2), the flat under-surface of the horizontal part of this measuring instrument is placed on the higher maxillary canine orthodontic bracket hook and held perpendicular to the true vertical (Fig. 3). The vertical ruler is held next to the contralateral canine tooth, and the vertical distance measured directly from the canine bracket to the flat under-surface of the horizontal part of the measuring instrument. Alternatively, if the patient is seated upright in the dental chair, the horizontal part of the instrument may be held parallel to the interpupillary line, assuming no vertical orbital dystopia is evident.

It is worth emphasising that the instrument may be held relative to a true vertical plumb line hanging from the ceiling if a patient has a severe craniofacial asymmetry and vertical orbital dystopia. However, this is usually not the case in orthognathic patients, and we suggest that when the interpupillary line is essentially parallel (i.e. the absence of vertical orbital dystopia), then the horizontal part of the instrument may be help parallel to the interpupillary line.

## Results

The vertical distance thus measured quantifies the overall extent of movement required to level a transversely canted maxillary occlusal plane.

## Discussion

One of the key principles in planning the correction of significant dentofacial asymmetries is levelling of the maxillary occlusal plane [1]. This decision is made primarily based on the aesthetic parameter of the maxillary incisor and canine exposure in relation to the upper lip in repose, and the degree and symmetry of the exposure of the maxillary dentition and gingivae in animation [1, 2]. If incisor and canine exposure is reduced, unilateral setdown of the maxilla may be required, albeit bearing in mind lower face height proportion and implications for surgical stability. Conversely, if dentogingival exposure is increased unilaterally, then ipsilateral maxillary impaction is the treatment of choice. The degree of impaction versus setdown required to accurately level the maxillary occlusal plane while maintaining or improving dentogingival aesthetics requires accurate planning. In addition to the important clinical and cephalometric techniques required for precision, the simple chair side technique potentially improves visualisation and permits an additional verification to aid both diagnosis and treatment planning for such patients.

## Conclusions

This measuring instrument and simple chair side technique helps to quantify the overall extent of surgical levelling required and may be a useful additional technique in our clinical diagnostic armamentarium.
